# Screening for the anti-inflammation quality markers of Xiaojin Pills based on HPLC-MS/MS method, COX-2 inhibition test and protein interaction network

**DOI:** 10.1038/s41598-018-25582-7

**Published:** 2018-05-10

**Authors:** Xi Xiong, Ya-nan He, Bi Feng, Yuan Pan, Hai-zhu Zhang, Xiu-mei Ke, Yi Zhang, Ming Yang, Li Han, Ding-kun Zhang

**Affiliations:** 1 State Key Laboratory Breeding Base of Systematic Research, Development and Utilization of Chinese Medicine Resources, Chengdu University of TCM, Chengdu, P.R. China; 2 Analysis & testing center,Chengdu University of TCM, Chengdu, P.R. China; 3 Department of Pharmacy and Chemistry, Dali University, Dali, P.R. China; 4 Chengdu Institutes of Food and Drug Control, Chengdu, P.R. China; 5 Jiangxi University of TCM, Nanchang, P.R. China

## Abstract

Nowadays, breast disorders seriously affect women’s health in an increasing number. In China, Xiaojin Pills are commonly used in the treatment of breast diseases. Doctors have concluded that the combined use of Xiaojin Pills with conventional therapy can significantly improve the efficacy with fewer side effects. However, the prescription of Xiaojin Pills is complicated and their quality control methods cannot completely ensure the quality of Xiaojin Pills. On the basis of its mechanism, our study combined chemical evaluation and biological evaluation to identify the anti-inflammatory markers of Xiaojin Pills. In this manuscript, 13 compounds in Xiaojin Pills were quantified. At the same time, the cyclooxygenase-2 inhibition rates of different Xiaojin Pills were measured and the possible markers were screened by spectrum-effect relationship. Further, anti-inflammatory activities of markers were verified and protein interaction network was analyzed, identifying the components of Protocatechuate, Beta-Boswellic acid and Levistilide A as the anti-inflammatory quality markers of Xiaojin Pills. We hope our studies can provide a scientific theoretical basis for accurately quality control of Xiaojin Pills and reasonable suggestions for pharmaceutical companies and new ideas for the quality control of other medicines.

## Introduction

With the acceleration of life pace and dramatic changes in society and natural environment, breast disease has become one of the most important factors threatening women’s health^1,2^. Among them, hyperplasia of mammary gland (HMG) is the most common problem troubled middle-aged women for its high morbidity, and recurrence rate^3,4^. What’s more, the prevalence of breast cancer in cyclomastopathy patients is as high as 2–4 times in healthy individuals^5–8^. Although the etiology of HMG is still not fully clear and appropriate therapies are limited^9^, more and more evidences^10–13^ indicate that the high expression of cyclooxygenase-2 (COX-2) is closely related to the inflammatory response and cell carcinogenesis.

At present, although conventional hormone therapy has a certain effect on the treatment of HMG, it is easy to relapse, and lead to endocrine disorders. In order to improve the effectiveness and alleviate the side effects, Chinese clinicians often combined Western medicine and Traditional Chinese Medicine (TCM) to treat HMG. The first choice of Chinese patent medicine is Xiaojin Pills, a famous prescription with a history of 200 years^14^. Xiaojin Pills have definite effects of dispersing swelling, detumescence, promoting blood circulation, and relieving pain. The whole regulation, multi-target and multi-channel action model makes it have a better therapeutic advantage^2,15^. Meta-analysis of 858 cases showed that on the basis of conventional western medicine treatment, combined with Xiaojin Pills treated for HMG, the total effective rate and cure rate was significantly higher than that of the control group, and no obvious adverse reaction was found^16^. In addition, it was also found that Xiaojin Pills showed a good effect on prostatitis^17^, arthralgia, thyroid nodule, mammary cancer, thyroid cancer and other cancers^18–20^ and the alleviating effect on prostatitis was achieved by inhibiting the expression of COX-2^17,21^. These results provided an important basis for screening the quality markers (Q-markers) of Xiaojin Pills from the anti-inflammatory effect and mechanism.

Xiaojin Pills consist of 10 kinds of Chinese herbal medicine (CHM), including Moschus, Momordicae Semen, Aconiti Kusnezoffii Radix Cocta, Liquidambaris Resina, Olibanum, Myrrha, Faeces Trogopterori, Angelicae Sinensis Radix, Lumbricus rubellus and Fragrant Ink. The sources of these drugs are complex, including plant medicine, resin medicine, animal medicine and mineral medicine. Due to the reliable efficacy, it was first recorded in the 1977 edition of Chinese Pharmacopoeia (CP). However, the latest edition of CP only defined the lower limit of muscone to evaluate the effectiveness, and identified three diester diterpenoid alkaloids by TLC method to control toxicity. Muscone is recognized as a component of effective anti-inflammatory activity and the single indicator is obviously unable to evaluate its quality and clinical effects, thus, it is necessary to study and screen the anti-inflammatory components other than muscone.

In order to accurately control the quality of Chinese medicine and Chinese patent medicine, the concept of Q-markers was proposed by Prof. Liu Chang-xiao (China) in 2016^22^. Q-markers could meet the following basic conditions. They are the chemical substances inherent in the products or formed during processing, and closely related to the functional properties of traditional Chinese medicine, and have definite chemical structures. Meanwhile, they should be the substances can be qualitatively identified and quantitatively measured. Due to the inherent difficulty in characterizing all chemical constituents in botanical drugs, Botanical Drug Development Guidance (BDDG) for Industry by United States FDA’s in 2016 clearly suggested the relative content or activity can be characterized by bioassay. Based on this concept and anti-inflammatory mechanism of inhibiting the high expression of COX-2, we put forward a new idea to evaluate the anti-inflammatory activity of Xiaojin pill by COX-2 inhibition test and screen the anti-inflammatory substances.

**Figure 1:**
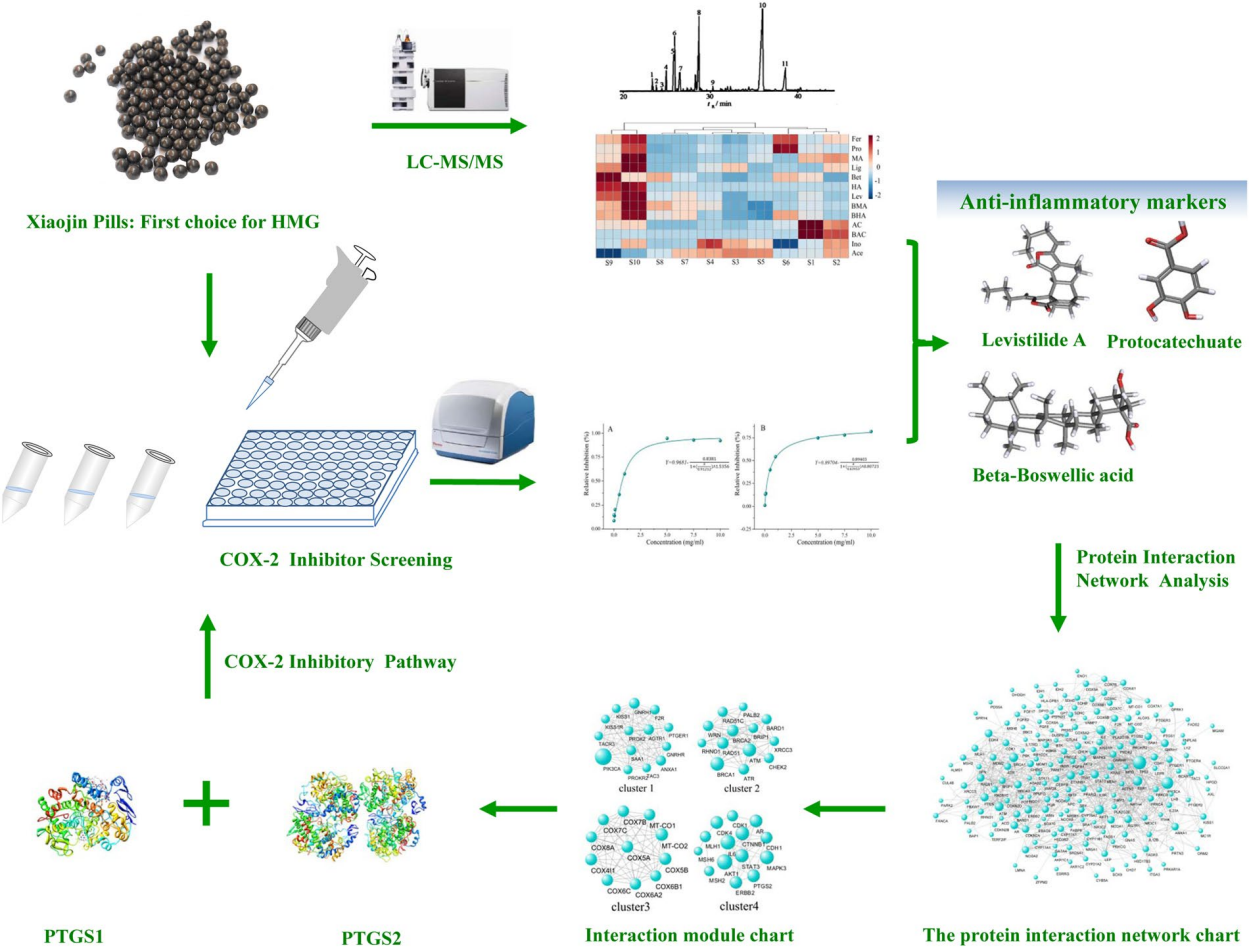
The screening process of Xiaojin Pills’ quality markers based on multi component content determination, COX-2 anti-inflammatory experiment and network interaction analysis.

In the present study, a rapid, sensitive, and accurate HPLC-MS/MS analytical method was developed to detected 13 kinds of candidate anti-inflammatory and analgesic components in Xiaojin Pills, including Aconitine (AC) Mesaconitine (MA), Hypaconitine (HA), Benzoylaconitine (BAC), Benzoylmesaconitine (BMA), Benzoylhypacoitine (BHA), Levistilide A (Lev), Inosine (Ino), Ligustilide (Lig), Acetyl-11-keto-b-boswellic acid (Ace), Beta-Boswellic acid (Bet), Ferulic Acid (Fer) and Protocatechuate (Pro). Validation parameters of the HPLC-MS/MS method were studied systematically^23^, and 10 batches of Xiaojin Pills were evaluated. Then, COX-2 inhibitor screening tests were carried out, and the correlation analysis was further performed to find possible anti-inflammatory ingredients. Finally, the screened markers were verified by COX-2 inhibition tests and protein interaction network analysis. Figure 1 showed the screening process of Xiaojin Pills’ quality markers. We hope that our study is benefit for the precise quality control and clinical application of Xiaojin Pills. The approach also provides examples for exploring the bioactive components of Chinese patent medicine and other ethnic drugs.

## Results

### Clustering analysis (CA) results

**Figure 2:**
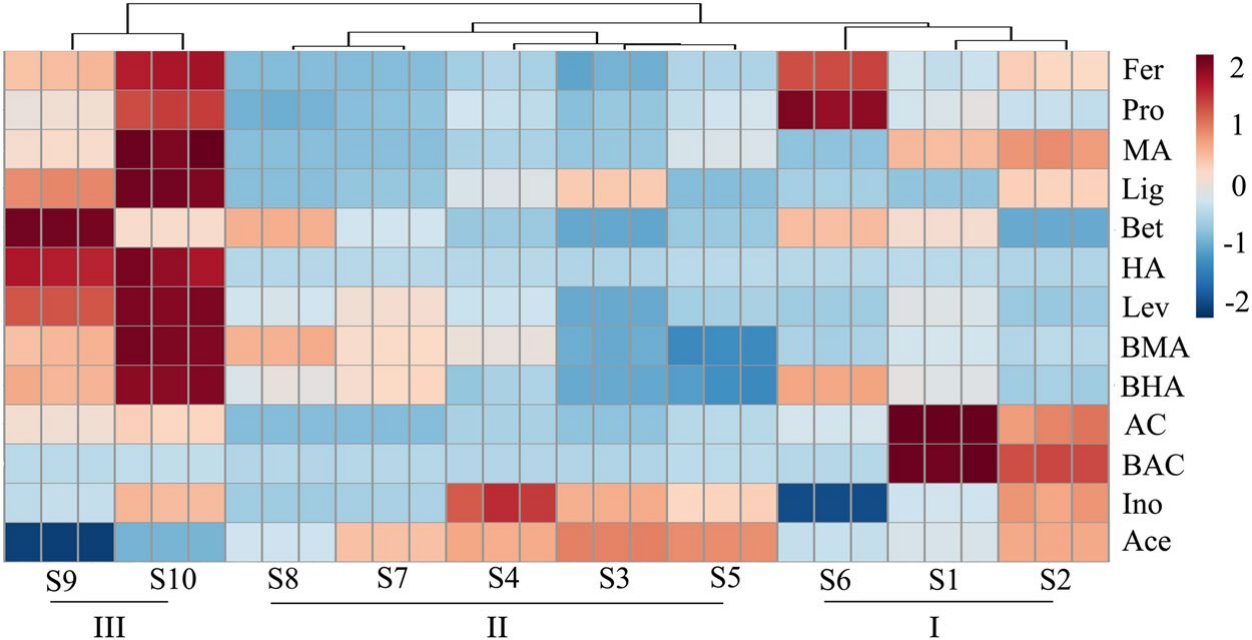
CA result of different batches of Xiaojin Pills based on chemical analysis.

The HPLC-MS/MS method was validated in terms of linearity, limit of detection (LOD) and limit of quantification (LOQ), precision, stability, repeatability and recovery based on ICH guidelines^24^. Their results were described in Supplementary Tables S1 and S2. The contents of 13 compounds in 10 samples of Xiaojin Pills were presented in Table S3. Contents of diester diterpenoid alkaloids in all samples were lower than 0.0031%, which showed that Xiaojin Pills were fairly safe. The other ten components differed greatly in different samples. To show the difference intuitively, the concentrations of thirteen components in 10 batches were used to create a 13 × 10 matrix where all the numerical values were displayed in terms of thermography (Fig. 2). Using an appropriate distance level, samples were obviously classified into three clusters. S1, S2 and S6 were categorized into cluster I. S5, S3, S4, S7 and S8 were grouped into cluster II. S9 and S10 were put into cluster III. It was clear that the contents of Fer, Pro, MA, Lig, Bet, HA, Lev, BMA and BHA in cluster III were obviously higher than others.

### Results of feasibility

**Figure 3:**
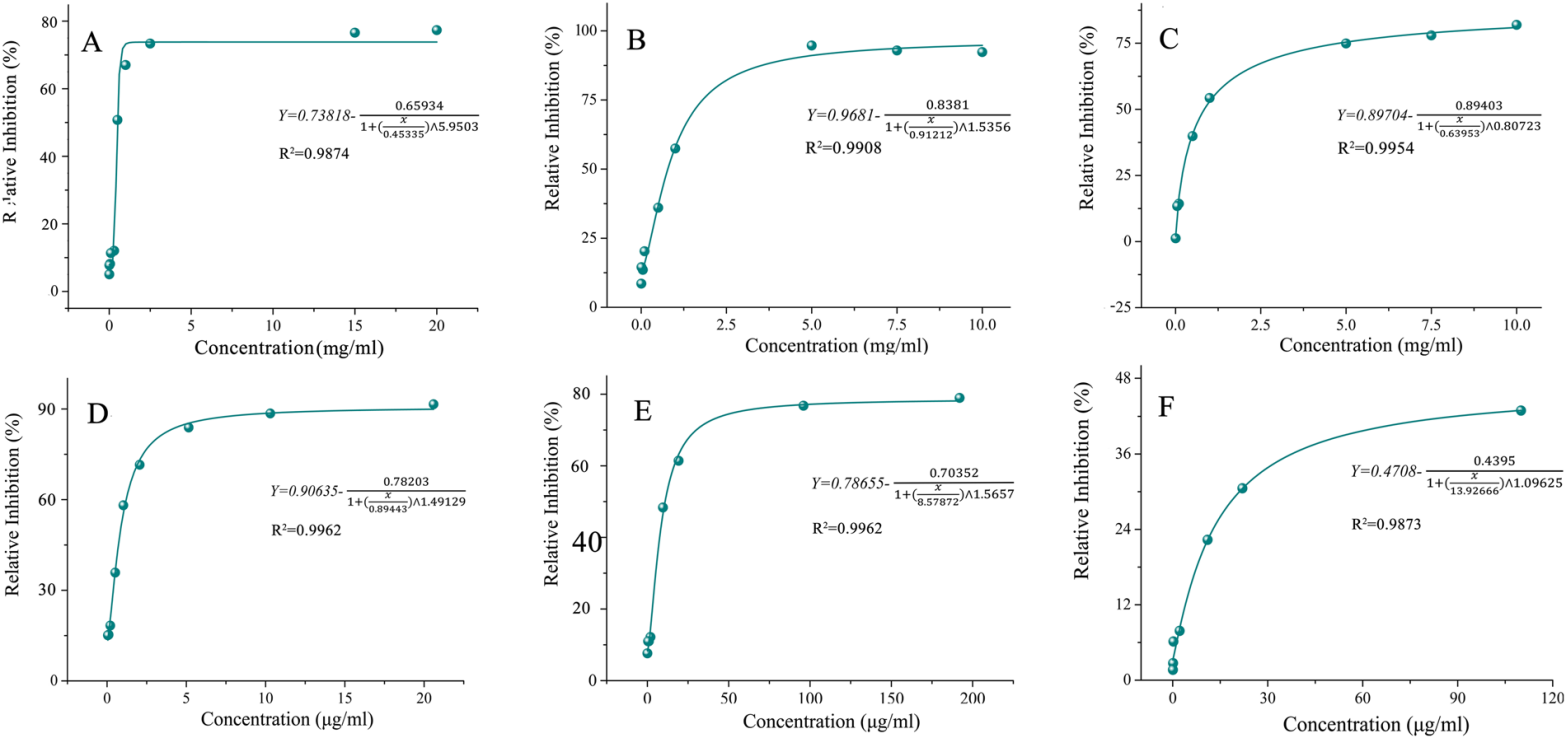
Concentration-COX-2 inhibition rate- curves of different Xiaojin Pills’ samples and three compounds. S4 (**A**), S1 (**B**), S6 (**C**), Pro (**D**), Bet (**E**) and Lev (**F**).

Results of inhibition curve (S4) were shown in Fig. 3A, and the *IC*_*50*_ was about 0.5076 mg/ml. At the concentration of 0.5 mg/ml, the actual inhibition rate of S1 and S6 was 34.04% and 37.64%, respectively. The inhibition rate curves of S1 and S6 were shown in Figs 3B and C. It was illustrated that the theoretical inhibition rate of S1 and S6 was 36.04%, and 39.86%, respectively. The error rate of S1 and S6 calculated was 5.55% and 5.57%, which indicated a direct determination method was of good feasibility.

### Results of COX-2 inhibition rate of samples

At the concentration of 0.5 mg/ml, the COX-2 inhibition rate of S1, S2, S3, S4, S5, S6, S7, S8, S9 and S10 were 34.25%, 28.12%, 21.66%, 47.04%, 44.08%, 37.64%, 43.47%, 52.07%, 51.72% and 35.91%, respectively.

### Results of regression analysis

Multiple linear regression analysis generalizes directly to multiple predictor variables and the multiple linear regression equation relates the continuous response variables (*Y*) to predictor variables (*x*). The regression equation was listed as:

1 $$Y=-\,{0.237852}+{0.013772}{x}_{1}+{0.000203}{x}_{2}+{0.014667}{x}_{3}$$

Among them, *Y* represented COX-2 inhibition rate, and *x*_1_, *x*_2_, *x*_3_ represented Pro, Bet and Lev, respectively.

The results showed that the F value was 37.974 and the corresponding P value was 0.000 (ie., less than 0.05), demonstrating that the multiple linear regression analysis model was satisfactory. It was found that a strong linear relationship between COX-2 inhibition rate and the contents of Pro, Bet and Lev.

The equation above described the degree which components in Xiaojin Pills contributed to the anti-inflammatory activity. Pro had the greatest important influence on COX-2 inhibition ratio, with correlation coefficient was 0.611. Bet and Lev were also positively correlated to inhibition ratio, and the correlation coefficients were 0.525 and 0.487, respectively. Therefore, Pro, Bet and Lev could be considered as the candidate anti-inflammatory markers.

### Results of anti-inflammatory activity of three components

Curve fitting method was used to evaluate the anti-inflammatory activity of three components. The results were shown in Fig. 3D–F. It was clear that the best inhibition rate of Pro was as high as 91.57%, while that of Bet and Lev was about 78.96% and 42.92%. It could be concluded that the anti-inflammatory inhibition activity of Pro was higher than Bet, and Lev, which was consistent with the correlation coefficient above.

### Results of protein interaction network

#### Compound target information

73 targets points were obtained by removing the repeated targets, including 45 of Pro, 33 of Bet, and 6 of Lev. From the number of targets, Pro contributed the most to the network. Among the targets we obtained, PTGS1 (prostaglandin 1/COX-1) and PTGS 2 (prostaglandin 2/COX-2) were the most important node in the process of prostaglandin metabolism. Therefore, Xiaojin Pills might be involved in the prostaglandin metabolic process to play a role in anti-inflammatory effect.

#### Construction of protein interaction and module analysis

**Figure 4:**
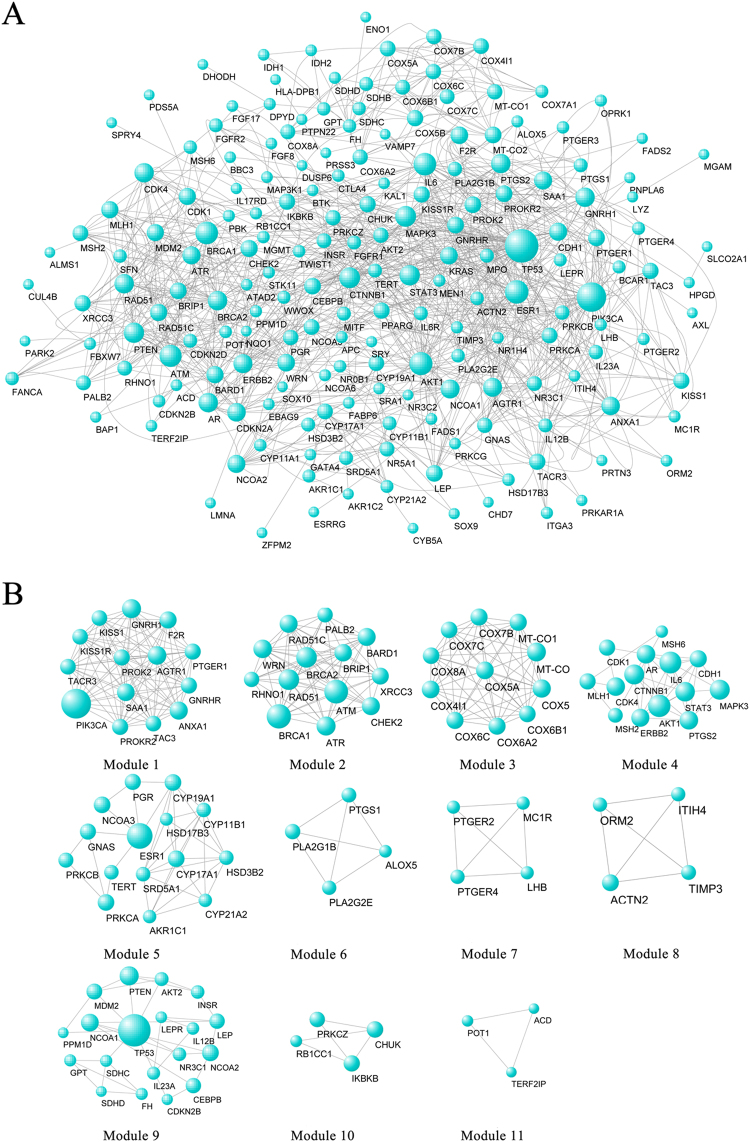
(**A**) The protein interaction network of Pro, Bet and Lev; (**B**) the modules of the protein interaction network of Pro, Bet and Lev.

Protein interaction information of targets from each compound was introduced into Cytoscape 2.8.3 and the protein interaction network diagram of Xiaojin Pills was obtained, including 186 nodes and 834 edges. Detailed information on these hubs is provided in Fig. 4A. Module analysis of protein interaction network had been conducted. As showed in Fig. 4B, 13 modules were identified. Using BinGo, the functions of the protein contained in the modules were annotated. Each module’s biological process was shown in Table 1. The module was graded according to the MCODE algorithm. The correlation of proteins in the modules became stronger as the score increased. Modules of 4th and 6th were mainly related to the prostaglandin metabolism process, including PTGS1 (prostaglandin peroxidase 1/COX-1), PTGS2 (prostaglandin peroxidase 2/COX-2). Prostaglandin (PG) was the metabolites produced by PTGS catalytic arachidonic acid. The PG synthesis regulated by PTGS was considered to be a marker of proinflammatory response and played an important role in the initiation of inflammation. The 10th module also played a role in inflammation, and the proteins it contained was related to the leukocyte chemotaxis mediated by chemotactic factors. This module included RB1CC1, PRKCZ, CHUK and IKBKB. Chemokines could cause an inflammatory reaction by inducing leukocytes to express integrins, releasing cytokines, inducing endothelial cells to express adhesion molecules, and collecting more inflammatory cells through the wall of vessels.

**Table 1:** The main biological process of the modules.

Cluster	Score	P-value	KEGG pathways
1	14	2.6 × 10^−6^	Neuroactive liqand-receptor interaction
2	12	1.3 × 10^−11^	Fanconi anemia pathway
3	11	9.8 × 10^−7^	Prostanoid metabolic process
4	5.385	1.2 × 10^−14^	Pathways in cancer
5	5	6.6 × 10^−12^	Steroid hormone biosynthesis
6	4	1.0 × 10^−5^	Arachidonic acid metabolism
7	4	6.4 × 10^−5^	Inflammatory mediator regulation of TRP channels
8	4	2.9 × 10^−6^	Platelet activation, signaling and aggregation
9	3.556	2.9 × 10^−6^	Central carbon metabolism in cancer
10	3.333	7.2 × 10^−4^	Chemokine signaling pathway
11	3	3.2 × 10^−5^	Cell cycle

During three anti-inflammatory markers screened, Pro and Bet both acted on COX-1 and COX-2, which were directly involved in the arachidonic acid metabolism pathway. However, it was not found a target directly related to this pathway associated with Lev. Through the analysis of protein interaction network, it was predicted that the inhibition effect of Pro and Bet on COX-2 was better than that of Lev.

## Discussions and Conclusions

During the study, different chromatography conditions were examined and compared, including various columns, mobile phases, and gradient elution conditions to achieve good resolution, high detection sensitivity, symmetric peak shapes, and short run time. Three kinds of reversed-phase columns, Kromasil C18 column (4.6 mm × 200 mm, 5 μm), Phenomenonex Gemini C18 column (4.6 mm × 150 mm, 5 μm) and Agilent Technologies Zorbax Eclipse XDB-C18 (4.6 mm × 150 mm, 5 μm), were investigated. The Agilent Technologies Zorbax Eclipse XDB-C18 column had good peak separation and sharp peaks. Poor resolution among the peaks was found when methanol was used as the organic solvent of the mobile phase. However, when methanol was replaced by acetonitrile, the resolution was greatly improved. Different concentrations of formic acid (0.05%, 0.1%, and 0.2%) or acetic acid (0.05%, 0.1%, and 0.2%) were added to the aqueous phase, respectively. 0.1% formic acid was selected with the best response and peak shape. The overall chromatographic run time was 30 min. The column temperature was set at 30 °C and the flow rate was 0.3 mL·min^−1^ to ensure good separation.

Mass spectral conditions were optimized in MRM scan type using the reference compounds. LC-MS/MS data of samples were collected by using an Agilent Triple Quadrupole mass spectrometer with an electrospray interface (ESI) operated both in positive and negative modes. The mode of the ESI was depended on the properties of compounds in gaining or losing electrons^25^. In order to get good response, AC, MA, HA, BAC, BMA, BHA, Lev, Ino and Lig were detected by the ESI^+^ mode, while Ace, Bet, Fer and Pro were detected by the ESI^−^ mode. In MRM mode, all compounds could be detected in different channels without interference. In addition, the temperature of 35 °C showed the highest extraction efficiency. For 0.2 g powder, 5 ml methanol was shown to be the optimal volume. The extraction time was studied in the range of 20 to 50 min. Finally, 30 min showed the highest extraction efficiency. As a result, the optimum conditions were: temperature of 35 °C, 5 ml methanol to 0.2 g powder and the extraction time of 30 min.

COX, also known as prostaglandin-endoperoxide synthase (PTGS), is an enzyme responsible for the formation of important biological mediators, including prostaglandins, prostacyclin and thromboxane. COX is the central enzyme in the biosynthetic pathway to prostanoids from arachidonic acid. COX-2 is one of the known isoenzymes, which is not expressed on normal conditions in most cells, but elevated levels are found during inflammation. Pharmacological inhibition of COX by non-steroidal anti-inflammatory drugs (NSAID) can provide relief from symptoms of inflammation and pain^10,11^. The experiment indicated that Xiaojin Pills played good anti-inflammatory and analgesic effects of reducing the expression of COX-2^21^.

The quality of Chinese patent medicine is mainly evaluated by the contents of active ingredients. However, it is a huge challenge to select the quality markers accurately for the complex effective material basis of patent medicine. In this study, a method of screening anti-inflammation markers based on spectrum-effect relationship has been practiced and the results provide a scientific basis for the formulation of quality control indicators. We believe our methods could provide a significant way to combine chemical methods and bioactivity evaluation to screen the biological activity markers in Chinese patent medicines or other valuable materials. It will be of interest for pharmaceutical companies and pharmaceutical workers.

## Materials and Methods

### Materials

Ten batches of Xiaojin Pills samples were collected from four pharmaceutical factories and all the information was listed in Table 2.

**Table 2:** Sample information.

Sample no.	Pharmaceutical factory	Batch no.	Production date
S1	Jiuzhaigou, Sichuan	151201	December 1, 2015
S2	Jiuzhaigou, Sichuan	150905	September 24, 2015
S3	Jiuzhitang, Schuan	160601	June 2, 2016
S4	Jiuzhitang, Schuan	151102	November 16, 2015
S5	Jiuzhitang, Schuan	150601.1	June 8, 2015
S6	Kaijing, Sichuan	20151003	October 28, 2015
S7	Kaijing, Sichuan	20160501	May 1, 2016
S8	Kaijing, Sichuan	20160502	May 2, 2016
S9	Yongkang, Sichuan	151009NO.015	November 4, 2015
S10	Yongkang, Sichuan	160806NO.073	November 3, 2015

HPLC-grade acetonitrile and methanol were purchased from Fisher Chemical (Pittsburg, PA, USA). HPLC-grade formic acid, ammonium acetate, and analytical-grade dimethyl sulfoxide (DMSO) were purchased from Chengdu KeLong Chemical Factory (Chengdu, China). The ultrapure water was obtained by Millipore Milli-Q water purification system (Millipore, Billerica, MA, USA). All solutions were filtered through 0.22 μm membranes (Jinteng, Tianjin, China) and degassed by ultrasonic bath before use. Screening kits for COX-2 inhibitors were purchased from Beyotiome Biotechnology (Shanghai, China). The fluorescence values were measured by Fluorescence microplate reader (GEMINIXS, USA) and the SOFTmaxPRO software was the production of Molecular Devices Company in USA.

Standards of MA(MUST-16032504), AC (MUST-16062206), HA (MUST-16032106), BMA (CHB151203), BAC (CHB160912), BHA (CHB160326) and Bet (CHB 170801) were purchased from Chroma-Biotechnology Co., Ltd (Chengdu, China). Ace (PRF8051801), Pro (15121808), Fer (PRF7101144) and Lig (PRF 15092501) were purchased from Chengdu Biopurify Phytochemicals Ltd (Chengdu, China). Lev (PSO756-0010) was obtained from Chengdu PUSH Bio-Technology Co., Ltd (Chengdu, China). Ino (151014) was a gift from Chengdu PureChem-Standard Co., Ltd (Chengdu, China). The purity of all standards is more than 98%.

### HPLC-MS/MS analysis

#### MS Conditions

Samples were analyzed by an Agilent1260 high performance liquid chromatograph and Agilent6460C triple-quadrupole tandem mass spectrometry (Agilent Technologies, Santa Clara, CA, USA) using an Agilent Technologies Zorbax Eclipse XDB-C18 column (4.6 mm × 150 mm, 5 μm). The column temperature was 30 °C and 0.1 μL of the sample solution was injected into the system. The mobile phase was composed of (A) 0.1% aqueous formic acid in water (ESI^+^ mode) or 10 mmol/L ammonium acid (ESI^−^ mode) and (B) acetonitrile using a gradient program of 100–80% B for 0–1 min, 80–50% B for 1–2 min, 50–30% B for 2–3 min, 30–80% B for 3–5 min, 80–100% B for 5–30 min, with a mobile flow rate of 0.3 mL/min.

**Figure 5:**
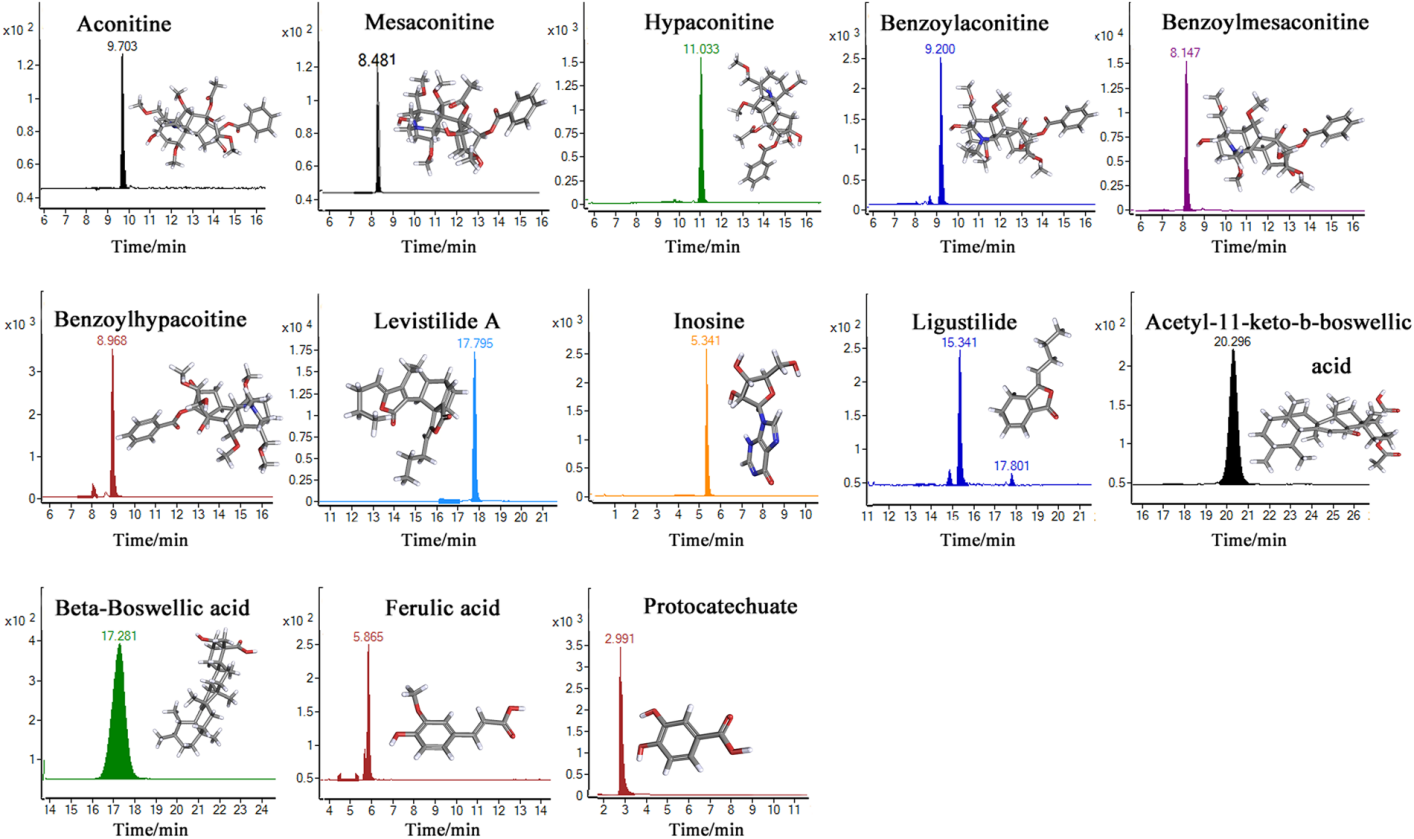
Typical MRM and chemical structures of the 13 compounds in Xiaojin Pills.

Mass spectrometric scan were obtained by ESI in ESI^+^ mode and ESI^−^ mode with a scanning interval 100–1000 m/z. The main parameters for MS were set as follows: gas temperature, 300 °C; gas flow, 11 L/min; nebulizer, 35 psig; capillary voltage, 4000 V; atomizer pressure 15 psi (1 psi = 6.895 Kpa). MS parameters and MRM transitions of each analyte are shown in Table 3. Based on this condition, the MRM chromatogram and chemical structure of each compound are shown in Fig. 5.

**Tablet 3:** Summary of molecular weight, multiple-reaction monitoring transitions, DP and CE of the 13 components determined by HPLC-MS/MS.

Compound	Molecular weight	Monitoring ion	Precursor ion (m/z)	Product ion (m/z)	DP (V)	CE (eV)
AC	645.7	ESI^+^	646.3	105.1	180	50
MA	631.7	ESI^+^	632.2	105.1	180	46
HA	615.7	ESI^+^	616.3	105.0	180	46
BAC	603.7	ESI^+^	604.3	105.0	180	45
BMA	589.7	ESI^+^	590.3	105.0	205	46
BHA	573.3	ESI^+^	574.3	105.0	200	46
Lev	380.2	ESI^+^	381.0	191.0	90	10
Ino	268.2	ESI^+^	268.9	136.9	80	5
Lig	190.2	ESI^+^	191.0	90.9	100	15
Ace	512.4	ESI^−^	511.3	59.1	110	12
Bet	456.4	ESI^−^	455.3	377.4	110	48
Fer	194.1	ESI^−^	193.0	133.8	100	12
Pro	154.0	ESI^−^	153.0	109.0	90	12

#### Preparation of standard solutions

The mixed standard containing 1.27 μg/mL AC, 1.02 μg/mL MA, 0.97 μg/mL HA, 1.12 μg/mL BAC, 1.03 μg/mL BMA, 0.90 μg/mL BHA, 0.96 μg/mL Lev, 1.03 μg/mL Ino, 0.998 μg/mL Lig, 1.013 μg/mL Fer, 0.98 μg/mL Pro, 1.09 μg/mL Ace and 0.96 μg/mL Bet was prepared stock into a volumetric flask and dissolved with 10 mL methanol. These solutions were stored in dark glass bottles at 4 °C and stable for at least 1 week. Working standard solutions were freshly prepared by diluting suitable amounts of the above solutions with methanol before injection.

#### Preparation of sample solutions

Break different batch of Xiaojin Pills to powder through a 50-mesh sieve. A total of 0.2 g of powder was accurately weighed and extracted with 5 mL of methanol solution by ultrasonic extraction for 30 min. Extracted solution was cooled, contributed to weight loss during the extraction procedure, and filtered through a 0.22 μm micropore film to yield the subsequent filtrate solution.

#### Determination of the sample

10 batches of Xiaojin Pills were determined by the method above.

#### CA

CA is a multivariate analysis method that is used to sort samples into groups^26^. In the present study, the CA of samples was performed using Metabo Analyst 3.0. Ward’s method as the amalgamation rule and squared Euclidean distance as metric were used to establish clusters. To visualize the similarity and dissimilarity of the samples, all the data were expressed as a thermograph.

### COX-2 inhibition rate measurement

#### Sample preparation

An aliquot of 0.2 g Xiaojin Pills powder (through a 50-mesh sieve) was accurately weighed and transferred into a 15-ml-flask. After being added with 10 mL of DMSO, the flask was weighed and extracted by ultrasonic extraction for 30 min. Extracted solution was cooled and the lost weight was supplied. Then the mixture was centrifuged at 4000 rpm for 10 min, the supernatant was transferred into another 15-ml-flask and the high concentration sample solutions for each batch were obtained.

#### Determination of suitable sample concentration

It is difficult to complete inhibition curve for each sample and calculate the *IC*_*50*_ value for the large amounts. Therefore, one sample (S4) was selected to calculate the *IC*_*50*_ value randomly, and it was used as a reference concentration. Firstly, S4 was prepared into solutions with a concentration of 20 mg/ml, 15 mg/ml, 0.5 mg/ml, 0.3 mg/ml, 0.1 mg/ml, 0.05 mg/ml, 0.01 mg/ml, and 0.005 mg/ml, respectively. Then, the inhibition rate of COX-2 in different concentration samples was measured and the inhibition curve was plotted. *IC*_*50*_ of S4 was calculated to be 0.5076 mg/ml. Based on it, each sample was diluted to a concentration of 0.5 mg/ml to determine the COX-2 inhibition rate.

#### Experimental procedure

According to the instructions of COX-2 inhibitor screening kit, the relative fluorescence unit (RFU) was measured after a period of incubation. Excitation wavelength was 560 nm, and emission wavelength was 590 nm. Inhibition rate of each sample was calculated as follows:

2 $$Inhibition\,rate\,( \% )=\frac{RF{U}_{2}-RF{U}_{S}}{RF{U}_{2}-RF{U}_{1}}\times {100} \% $$

Among them, RFU_1_, RFU_2_, and RFU_S_ represented the fluorescence value of the blank control group, 100% enzyme activity control group and the sample group, respectively.

#### Feasibility verification

S1 and S6 were randomly selected to compare the results difference between direct determination method and curve fitting method. The former was determined at the fixed concentration of 0.5 mg/ml. It was considered as an actual value. The later was calculated by the best fitted curve equation after the study of relationship between concentrations and inhibition rates. This result was accepted as a theoretical value. Error rate of direct determination method was calculated by formula 2.

3 $$Error\,rate\,( \% )=\frac{I{R}_{a}-I{R}_{t}}{I{R}_{t}}\times {100} \% $$

Among them, IR_a_, IR_t_ represented the fluorescence value of the actual inhibition rate and the theoretical inhibition rate, respectively.

#### Multiple linear regression analysis

Multiple linear regression analysis was used to model the best combination of two or more independent variables (*x*_*i*_) to predict or estimate the dependent variable (*Y*) by fitting a linear equation^27^. It showed the contribution of each independent variable to the dependent variable in the following form:

4 $$Y={b}_{{0}}+\sum _{i=1}^{n}bixi\,(n=1,\,2,\,3,\,4\ldots )$$

where Y was the estimated value and represents the dependent variable and *x*_*i*_ were the uncorrelated variables; b_0_ represented the estimated constant, and *b*_*i*_ was regression coefficients. In this study, multiple linear regression analysis was introduced to combine data from the chemical contents determination and anti-inflammatory activity of Xiaojin Pills. SPSS statistical software (SPSS for Windows 13.0, SPSS Inc., USA) was used to establish the chemical-activity relationships and further explore the anti-inflammatory markers.

#### Verification of anti-inflammation markers

The anti-inflammation activities of the markers we screened were compared by the curve fitting method. After the study of relationship between concentrations and inhibition rates, several best fitted curves of the markers were obtained respectively, and the anti-inflammation effects of several markers could be verified by comparing their best inhibition rates.

### Protein interaction network

#### Collection of the potential targets

Target data of the anti-inflammatory markers were excavated and the target information of candidate components were derived from STITCH (http://stitch.em-bl.de/), BATMAN-TCM (http://bionet.ncpsb.org/batman-tcm), TCMID (https://omictools.com/traditio), TCMSP (http://ibts.hkbu.edu.hk/LSP/tc) and ChEMBL (https://www.ebi.ac.uk/chembl/). STITCH database could generate a score for each component target relationship, which ones with high confidence data (score >0.7) was approved to ensure the reliability of data^28^.

#### Construction and analysis of protein interaction network

The protein interaction information of the target was derived from the String database (http://www.string-db.org/). The protein interaction data we obtained were introduced into Cytoscape 2.8.3 and each of the target protein interaction networks was calculated by the Union method^13,29^. After removing the isolated points, repeated and self-loop edges, we got the interaction network of anti-inflammatory markers.

#### Pathway enrichment analysis

Because of the great number of compounds of an herb and the multiple targets of each compound, the total number of the targets of all the key herbs is substantial. The great amount of targets makes it difficult to understand the biological meaning, so a pathway enrichment analysis was performed using the Database Visualization and Integrated Discovery software (DAVID, http://david.abcc.ncifcrf.gov/home.jsp, version 6.7.) and based on the pathway data obtained from the Kyoto Encyclopedia of Genes and Genomes database (KEGG, http://www.genome.jp/kegg/, updated on April 18, 2016)^30^.

## Electronic supplementary material

Supplementary information
