# Supplementary material for: Screening for the anti-inflammation quality markers of Xiaojin Pills based on HPLC-MS/MS method, COX-2 inhibition test and protein interaction network
Source: Sci Rep. 2018 May 10;8:7454. doi: 10.1038/s41598-018-25582-7 (PMC5945850; doi:10.1038/s41598-018-25582-7)
Supplement: Supplementary file 1 — Supplementary information [file 41598_2018_25582_MOESM1_ESM.doc]

**Screening for the anti-inflammation quality markers of Xiaojin Pills based on HPLC-MS/MS method, COX-2 inhibition test and** **protein interaction network**

Xi Xiong^1^, Ya-nan He^1^, Bi Feng^1^, Yuan Pan^2^, Hai-zhu Zhang^3^, Xiu-mei Ke^1^, Yi Zhang^4^, Ming Yang^5^, Li Han^1^*, Ding-kun Zhang ^1^*

*^1.^State Key Laboratory Breeding Base of Systematic Research, Development and Utilization of*

*Chinese Medicine Resources, Chengdu University of TCM, Chengdu, PR China*

*^2.^Analysis & testing center,Chengdu University of TCM, Chengdu, PR China*

*^3.^Department of Pharmacy and Chemistry, Dali University, Dali, PR China*

*^4.^Chengdu Institutes of Food and Drug Control, Chengdu, PR China*

*^5.^Jiangxi University of TCM, Nanchang, PR China*

**Corresponding author: Dr. Ding-kun Zhang, E-mail: 465790643@qq.com;*

*Professor. Li Han, E-mail: hanliyx@163.com*

**Supplementary Information**

**Table S1**. Regression equations, correlation coefficients, linear ranges, LOD and LOQ of 13 compounds

| Compound | Regression equation | r | Liner range  (μg·mL^-1^) | LOQ  (ng•mL^-1^) | LOD  (ng•mL^-1^) |
| --- | --- | --- | --- | --- | --- |
| AC | *y*=2145.4*x*+36.095 | 0.9979 | 0.0127~2.5400 | 0.79 | 3.20 |
| MA | *y*=5607.3*x*+257.41 | 0.9990 | 0.0515~10.3000 | 0.77 | 2.91 |
| HA | *y*=24474*x*+275.3 | 0.9990 | 0.0024~0.2425 | 0.70 | 2.70 |
| BAC | *y*=119045*x*-2953.6 | 0.9989 | 0.0560~11.2000 | 0.71 | 2.91 |
| BMA | *y*=27340*x*-17448 | 0.9990 | 0.0155~3.0900 | 0.74 | 3.09 |
| BHA | *y*=74457*x*-16811 | 0.9986 | 0.0225~4.5000 | 0.025 | 10.167 |
| Lev | *y*=456.7*x*+1273.4 | 0.9995 | 0.0024~0.4800 | 0.75 | 2.4 |
| Ino | *y*=13773*x*+1292.9 | 0.9989 | 0.0206~4.1200 | 10.3 | 25.75 |
| Lig | *y*=1489.6*x*+348.78 | 0.9979 | 0.0399~7.9840 | 3.99 | 15.9 |
| Ace | *y*=960.23*x*+2093.1 | 0.9999 | 1.6600~261.6000 | 10.9 | 21.0 |
| Bet | *y*=149.82*x*+0.9763 | 0.9996 | 0.1920~9.6000 | 9.6 | 18.2 |
| Fer | *y*=809.46*x*-844.63 | 0.9979 | 0.4025~10.1300 | 5.05 | 10.13 |
| Pro | *y*=27059*x*-30549 | 0.9997 | 0.9600~78.4000 | 1.01 | 11.3 |

**Table S2**. Results of precision, stability, repeatability and recovery

| Compound | Precision RSD/% | Stability RSD/% | Mean mass fraction/(μg·g^-1^) | Repeatability RSD/% | Recovery  Mean% RSD% | |
| --- | --- | --- | --- | --- | --- | --- |
| AC | 3.3640 | 3.2928 | 6.46689 | 4.5694 | 97.9152 | 4.4272 |
| MA | 0.4659 | 4.2232 | 3.039625 | 2.4692 | 101.0089 | 1.7854 |
| HA | 0.9975 | 3.0929 | 1.659989 | 3.5398 | 94.8169 | 1.5827 |
| BAC | 2.1764 | 1.7638 | 2.633601 | 1.1302 | 101.3509 | 3.8486 |
| BMA | 0.8331 | 1.1637 | 44.33336 | 1.4464 | 93.9479 | 1.4527 |
| BHA | 0.9154 | 0.7265 | 8.60241 | 1.1302 | 97.6298 | 3.5634 |
| Lev | 3.3876 | 2.5649 | 8.263625 | 4.0209 | 98.4893 | 1.2965 |
| Ino | 0.8564 | 2.9342 | 14.24125 | 1.2889 | 104.3326 | 3.9750 |
| Lig | 0.9975 | 2.9542 | 136.7232 | 3.5398 | 94.8149 | 1.5823 |
| Ace | 0.2443 | 2.1030 | 777.9819 | 2.3147 | 96.7938 | 2.2396 |
| Bet | 0.7899 | 4.4444 | 311.1656 | 3.6545 | 96.5429 | 2.0098 |
| Fer | 2.9372 | 2.0542 | 34.43794 | 1.0415 | 95.7964 | 2.3422 |
| Pro | 3.3840 | 0.8219 | 31.04584 | 0.5876 | 96.9143 | 4.6163 |

**Table S3**. Contents of 13 compounds in 10 samples of Xiaojin Pills (n=3)

| S | Content of each compound in 10 batches of Xiaojin Pills sample (μg/g) | | | | | | | | | | | | |
| --- | --- | --- | --- | --- | --- | --- | --- | --- | --- | --- | --- | --- | --- |
|  | AC | MA | HA | BAC | BMA | BHA | Lev | Ino | Lig | Ace | Bet | Fer | Pro |
| S1 | 28.36 | 2.27 | 0.13 | 68.12 | 32.54 | 7.75 | 3.86 | 16.15 | 26.46 | 1486.62 | 488.84 | 27.08 | 29.47 |
| S2 | 19.76 | 3.28 | 0 | 55.82 | 33.67 | 7.20 | 1.75 | 29.29 | 114.33 | 2057.63 | 197.50 | 40.80 | 31.07 |
| S3 | 1.23 | 0.28 | 0 | 1.14 | 29.38 | 7.37 | 0 | 35.48 | 154.80 | 2751.33 | 247.48 | 27.24 | 32.12 |
| S4 | 4.10 | 0.70 | 0.15 | 1.89 | 53.80 | 9.56 | 4.50 | 46.72 | 100.26 | 2626.30 | 394.40 | 35.26 | 39.51 |
| S5 | 5.68 | 1.63 | 0.18 | 4.64 | 20.77 | 6.57 | 2.76 | 30.63 | 33.28 | 2693.27 | 384.30 | 35.22 | 40.95 |
| S6 | 3.85 | 0 | 0 | 1.17 | 17.42 | 6.51 | 1.06 | 0.98 | 23.43 | 930.42 | 379.97 | 32.20 | 37.08 |
| S7 | 0.58 | 0 | 0.18 | 2.48 | 62.64 | 13.72 | 7.89 | 21.41 | 44.44 | 2660.78 | 605.52 | 31.26 | 33.49 |
| S8 | 0.60 | 0 | 0.13 | 2.39 | 77.71 | 12.91 | 5.48 | 21.15 | 37.41 | 2382.94 | 1024.40 | 32.70 | 31.83 |
| S9 | 8.40 | 1.64 | 2.29 | 2.54 | 44.36 | 9.39 | 9.86 | 14.82 | 135.17 | 843.64 | 1003.55 | 36.76 | 31.19 |
| S10 | 6.47 | 3.04 | 1.66 | 2.63 | 44.33 | 8.60 | 8.26 | 14.24 | 136.72 | 777.98 | 311.17 | 34.43 | 31.05 |
